# Feasibility of arterial spin labeling in evaluating high- and low-flow peripheral vascular malformations: a case series

**DOI:** 10.1259/bjrcr.20210083

**Published:** 2021-08-12

**Authors:** Sanjeev Ramachandran, Jonathan Delf, Christopher Kasap, William Adair, Harjeet Rayt, Matthew Bown, Neghal Kandiyil

**Affiliations:** 1 University Hospitals of Leicester NHS Trust, Infirmary Square, Leicester, United Kingdom; 2 Siemens Healthineers, Sir William Siemens Square, Frimley, Surrey, United Kingdom

## Abstract

We present a case series highlighting a novel use of arterial spin labeling (ASL), a MRI perfusion technique, to evaluate both high- and low-flow peripheral vascular malformations (PVMs) across a range of anatomical locations. While the role of ASL in assessing intracranial vascular malformations is more established, there is limited evidence for PVMs. Our results provide preliminary evidence for the feasibility of ASL in imaging PVMs and its potential ability to distinguish between high- and low-flow PVMs. In addition, we demonstrate its ability to identify focal high blood flow, which may indicate the nidus in arteriovenous malformations. Together, these findings have important implications for patient management. We also outline the potential benefits and limitations of ASL in the imaging of PVMs, and provide justification for further validation of its diagnostic performance.

## Introduction

Peripheral vascular malformations (PVMs) represent non-lethal failure of early embryonic vascular differentiation, presenting as a broad spectrum of vascular lesions. The classification system of the International Society for the Study of Vascular Anomalies (ISSVA) further divides these lesions by flow dynamics into high- or low-flow lesions.^1,2^ Furthermore, there is variation in the cellular phenotypes of PVMs, including lymphatic, venous, capillary, arterial, arteriovenous or mixed.^1,2^ The differentiation between high- and low-flow lesions and ascertaining their angioarchitectural features are essential in guiding management.^1,2^ High-flow PVMs usually require planning with catheter angiography and complex endovascular embolisation, whilst low-flow PVMs usually require relatively simple sclerotherapy.^2^ Therefore, distinguishing between the two reduces unnecessary intervention and multiple attendances to the hospital.

Imaging has an important role in the work-up of such lesions. Arterial spin labeling (ASL) is a MR perfusion technique where water in arterial blood is used as an endogenous freely diffusible tracer.^3^ This involves labelling inflowing arterial blood by applying an inversion radiofrequency pulse proximal to the imaging plane. Subsequently, signal from the arterial blood in the imaging plane is subtracted from the unlabelled control images, with the resultant signal intensity being proportional to blood flow.^4^

ASL imaging has an increasingly established role in the evaluation of intracranial arteriovenous malformations (AVMs).^5^ However, there is a relative paucity of evidence on the role of ASL in the imaging of PVMs. To date, there has been one case report highlighting the novel use of ASL in evaluating a forearm AVM,^6^ with a further study demonstrating its ability in discriminating between cervicofacial vascular malformations in paediatric patients by detecting differences in intralesional flow.^7^ The aim of this case series therefore is to further elucidate the role of ASL in PVM imaging, particularly in discriminating between high- and low-flow lesions.

## Methods

Patients were recruited from a specialist PVM clinic at a tertiary referral centre over a 6-month period (May–October 2019). Case selection was dependent on if patients already required MR imaging as part of their work-up during the defined time period. Exclusion criteria were based on any contraindications to MRI. Categorisation into high- and low-flow lesions was based on a combination of sonographic and MRI features, with corroboration from catheter angiography where there was still uncertainty. Interpretation of all imaging was achieved by consensus between two radiologists, one with expertise in vascular radiology. Ethical approval was not required as ASL is used as a routine investigation in our centre’s normal practice.

All patients presented underwent ASL imaging, with the majority also imaged using MR angiography (MRA) with an intravenous gadolinium bolus (3D time-resolved angiography with stochastic trajectories or TWIST) in addition to T1, T2 and fat suppression sequences. ASL imaging was performed prior to any gadolinium-enhanced MRA. The labelling slice was applied above the most proximal extent of the lesion. In line with the previous case report,^6^ 3D pulsed ASL employing a Turbo Gradient Spine Echo (TGSE) sequence was used with the following parameters: magnetic field strength 1.5 Tesla (MAGNETOM Aera, Siemens Healthineers, Erlangen, Germany), labelling (bolus) duration 700 ms, post-labelling delay time 1990 ms, flip angle 180^o^, TR 4600 ms, TE 20.44 ms, slice thickness 4 mm, interpolated voxel size 1.9 × 1.9 × 4 mm, base resolution 64 and phase resolution 97%. Image acquisition time varied between approximately 5–7 min depending on the number of slices per slab acquired. Scrollable grayscale maps of baseline magnetization were generated using inbuilt software from Siemens Healthineers (Syngo N4 VE11C), which was used for assessment of possible artefacts and technical error. These images were transferred to the PACS. The maps had full cross-referencing capabilities with 3D reformation to the anatomic MR images.

## Results

We present the imaging findings from a selection of three high-flow and three low-flow cases across different anatomical locations below.

## High flow

### Case 1

**Figure 1.**
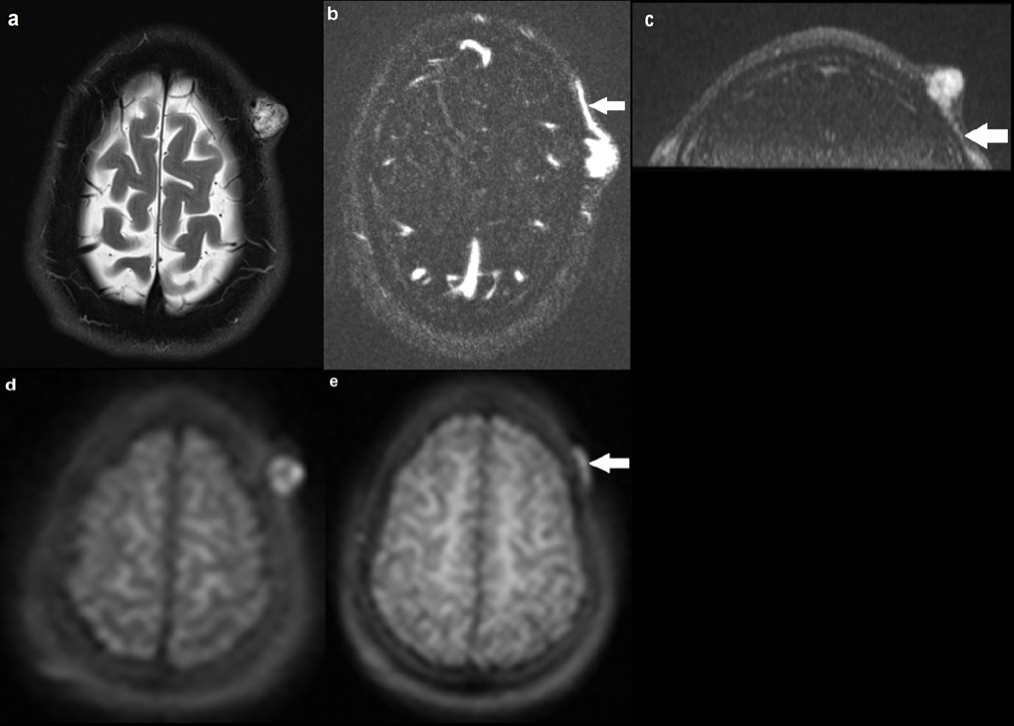
a) Axial T2 image demonstrating a 18 × 16 mm hyperintense subcutaneous lesion in the left frontal scalp region, with multiple flow voids within. Underlying calvarium is intact; (**b**) axial MRA (TWIST) demonstrating robust early (arterial phase) enhancement of the lesion, with a draining vein shown at the same level (solid arrow). No communication with the intracranial circulation; (**c**) coronal MRA delineating an arterial feeder arising from the left superficial temporal artery (solid arrow); (**d**) axial ASL image demonstrating hyperintensity within the lesion, suggestive of high flow; (**e**) axial ASL image at a different slice delineating the draining vein (solid arrow), with the arterial feeder seen on MRA not identified. ASL, arterial spin labeling.

A 26-year-old male presented with a pulsatile lesion overlying the left frontal bone (Figure 1). MRI revealed a small T1 hypointense and T2 hyperintense subcutaneous lesion in the left frontal scalp region with multiple tortuous flow voids within. Arterial feeders were seen to arise from the left superficial temporal artery with venous drainage into the superficial scalp veins. There was robust early (arterial phase) enhancement of the lesion on the dynamic MRA. The underlying calvarium was intact with no communication with the intracranial circulation. The appearances on the conventional MRI sequences were consistent with a high-flow arteriovenous haemangioma, or cirsoid aneurysm, of the scalp. This correlated to intralesional hyperintensity on the ASL images, in keeping with a high-flow lesion. In addition, the draining vein was identified on ASL but the arterial feeder was not convincingly demonstrated.

### Case 2

**Figure 2.**
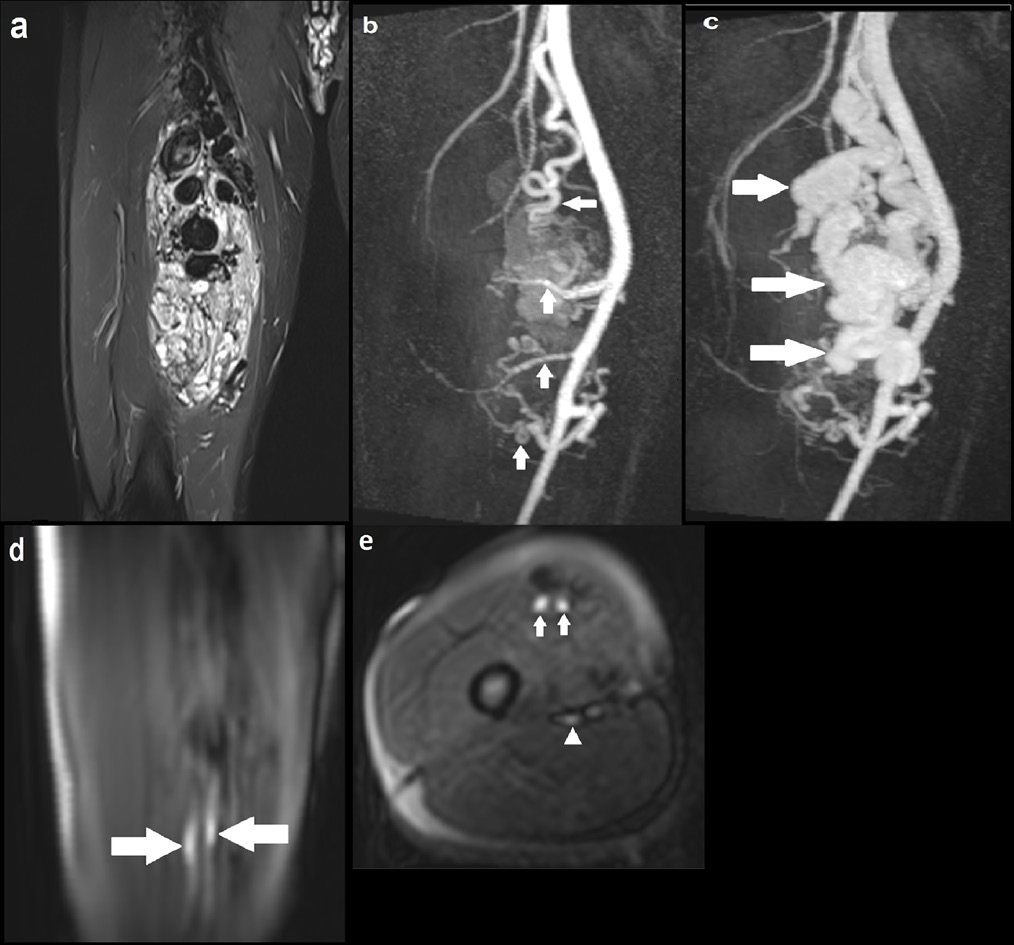
(a) Coronal STIR image demonstrating a large AVM with multiple large tortuous flow voids involving the vastus medialis and intermedius muscles; (**b**) coronal MIP image of dynamic MRA (TWIST) in the early arterial phase delineating four arterial feeders arising from the proximal, mid and distal SFA (solid arrows), with early enhancement of the AVM; (**c**) Subsequent MRA MIP image in the arterial phase demonstrating avid enhancement of the AVM with a large draining venous channel (solid arrows), multiple smaller venous channels and early filling of the distal SFV; (**d**) Coronal ASL image demonstrating two discrete foci of hyperintensity within the AVM (solid arrows), suggestive of the nidus through which blood flow is maximal; (**e**) Axial ASL image again highlighting the two foci of intense signal suggestive of the nidus (solid arrows). At the same level, there is another focus of less marked high signal posteriorly indicating early venous drainage (solid arrowhead); (**f**) Axial ASL image acquired at another slice identifying one of the superior arterial feeders (solid arrow). ASL, arterial spin labeling; AVM, arteriovenous malformation; MIP, maximum image projection; SFA, superficial artery; SFV, superficial vein; STIR, short tau inversion recovery.

A 28-year-old male presented with a pulsatile lump in the right thigh (Figure 2). Appearances on the conventional sequences were consistent with a large high-flow AVM involving the right vastus medialis and vastus intermedius muscles. MRA identified four main arterial braches of the right superficial artery (SFA) feeding into the AVM with one large and multiple small venous channels draining the lesion into the distal superficial femoral vein (SFV). The ASL images demonstrated two small discrete foci of intensely high signal within the lesion, which were thought to represent the nidus through which there was maximal blood flow. This was not distinguishable on the MRA images. Moreover, ASL identified early filling of the distal SFV consistent with arteriovenous shunting and some, but not all, arterial feeders.

### Case 3

**Figure 3.**
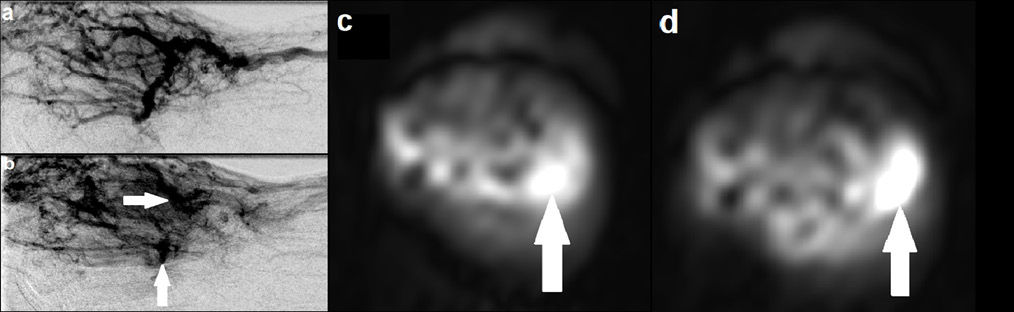
(a) Catheter angiogram demonstrating an AVM involving the right hand with evidence of arteriovenous shunting; (**b**) catheter angiogram at a slightly later time point identifying two discrete niduses (solid arrows); (**c**, d) Axial ASL images at different slices demonstrating two separate foci of intensely high signal (solid arrows), corresponding with the niduses seen on catheter angiogram and providing evidence of high flow. ASL, arterial spin labeling; AVM, arteriovenous malformation.

A 34-year-old female was under follow up for a known AVM in the right hand (Figure 3). A previously performed catheter angiogram demonstrated a high-flow lesion in the right hand with evidence of arteriovenous shunting, consistent with an AVM. Two separate niduses were identified with no target for embolisation. This correlated well to the ASL images, which demonstrated lesional hyperintensity consistent with high flow. In particular, there were two foci of intensely high signal corresponding to the niduses seen on catheter angiography. These were not identifiable on MRA.

## Low flow

### Case 4

**Figure 4.**
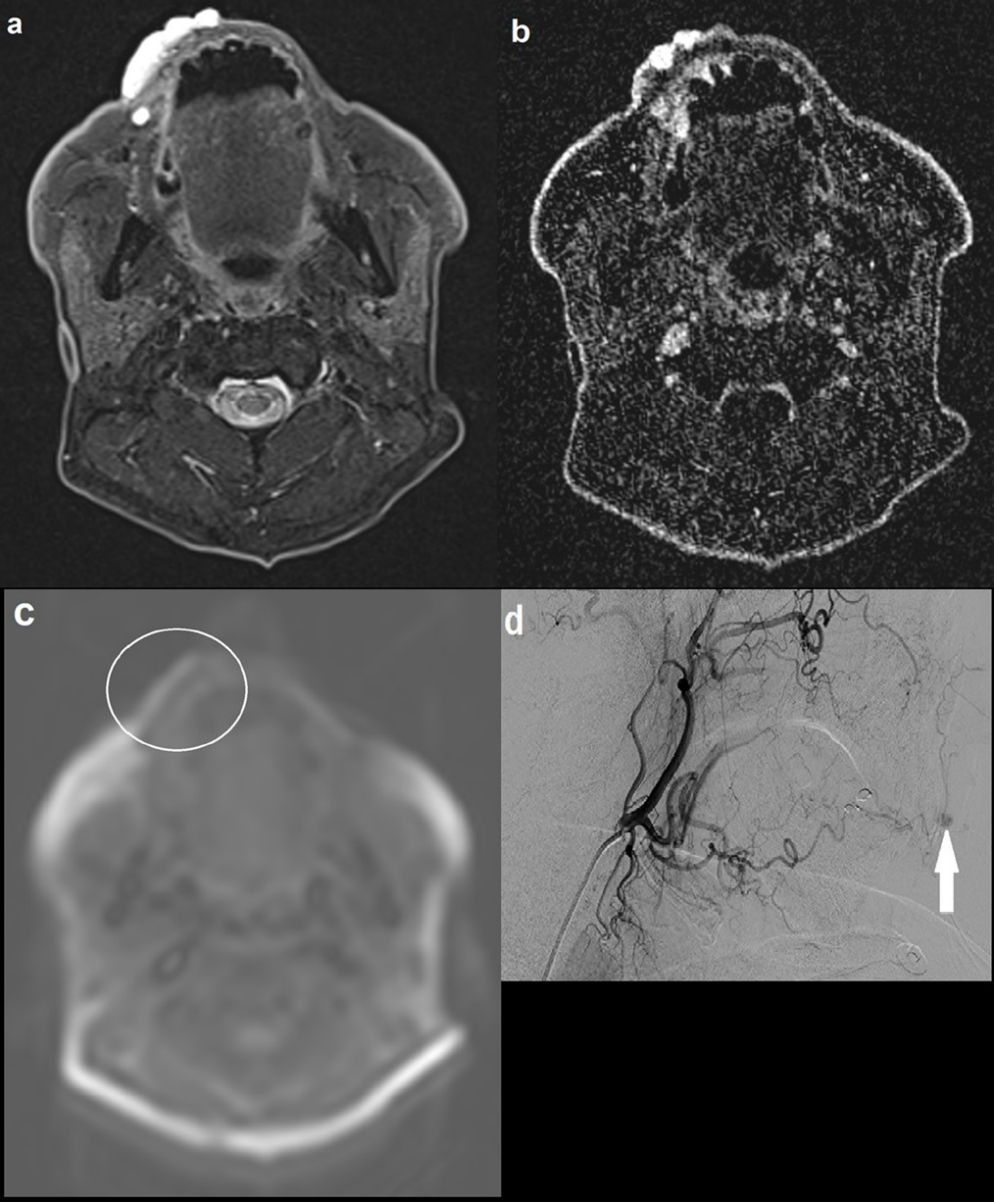
(a) Axial STIR image demonstrating a 4.2 cm hyperintense lesion involving the right upper lip; (**b**) axial MRA (TWIST) image at approximately the same level showing maximal enhancement in the late images, with no definite arterial feeders or draining veins; (**c**) axial ASL image demonstrating normal signal intensity within the lesion. There is artefactual high signal at the outer border of the lip (not corresponding to the lesion) and elsewhere at the edges of the image; (**d**) carotid catheter angiogram showing maximal contrast opacification of the lesion in the delayed phase (solid arrow), with capillary type feeding vessels arising from the lingual artery and no large draining vein. No communication with the intracranial circulation. Appearances were in keeping with a low-flow verrucous vascular malformation, thought to be a predominantly venous malformation. ASL, arterial spin labeling; STIR, short tau inversion recovery.

A 77-year-old male presented with a lesion on his upper right lip which was growing in size over the past year and bleeding (Figure 4). MRI demonstrated a T2 hyperintense and T1 isointense focus involving the upper right lip. There was maximal enhancement of the lesion in the delayed phase of the dynamic MRA with no identifiable feeding arteries or draining veins. ASL did not identify abnormal signal intensity within the lesion, suggestive of a low-flow PVM. This was corroborated by a subsequent carotid catheter angiogram, which found features indicative of a low-flow verrucous venous malformation with capillary type feeding vessels arising from the lingual artery and no large draining vein.

### Case 5

**Figure 5.**
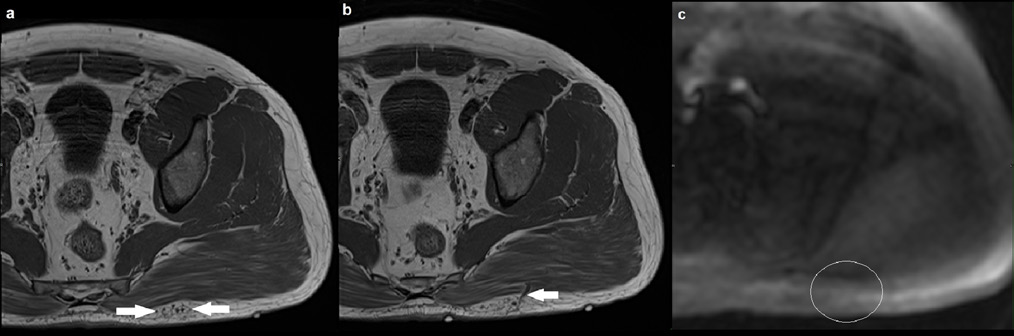
(a) Axial T1 image demonstrating a small cluster of veins in the superficial subcutaneous tissue overlying the left gluteus maximus muscle (solid arrow), with no intramuscular component. There was fat suppression in between the cluster of veins (not shown); (**b**) axial T1 image at a different level showing one of the two draining veins traversing the gluteus maximum muscle (solid arrow), going onto communicate with normal-sized gluteal veins; (**c**) axial ASL demonstrating normal signal intensity in the region of interest (outline by a white circle). There is artefactual high signal at the edges of the image. ASL, arterial spin labeling.

A 36-year-old male presented with an asymptomatic lump over his left buttock which was increasing in size (Figure 5). Conventional MRI sequences demonstrated a small cluster of veins in the superficial subcutaneous tissue, with two draining veins that traversed the left gluteus maximus muscle and eventually communicated with normal-sized gluteal veins. There were no suspicious features for malignancy. As clinical examination, sonographic findings and MR imaging features were all consistent with a low-flow PVM, it was decided that MRA would not add any further diagnostic information. There was corresponding normal signal intensity on ASL in the region of interest, consistent with a low-flow PVM.

### Case 6

**Figure 6.**
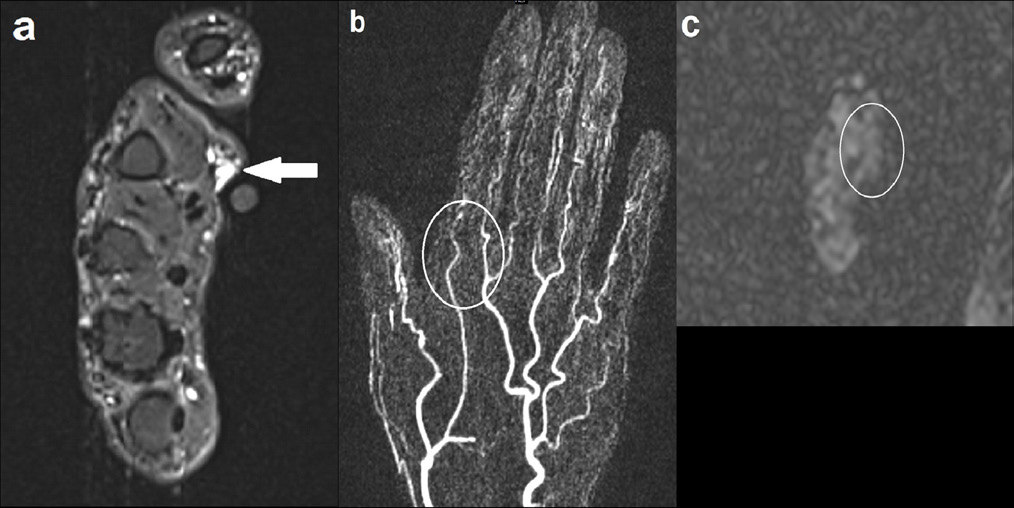
(a) Axial STIR image demonstrating a small cluster of vessels in the subcutaneous tissue overlying the palmar aspect of the second metacarpal head, measuring 7 × 9 mm (solid arrow). No involvement of the underlying musculature; (**b**) coronal MIP image of dynamic MRA (TWIST) at the level of the lesion demonstrating no enhancement in the early arterial phase or arterial feeders (outline by the white circle); (**c**) axial ASL showing normal signal intensity in the region of interest (outline by the white circle). ASL, arterial spin labeling; MIP, maximum image projection; MRA, MR angiography; STIR, short tau inversion recovery.

A 57-year-old female presented with a lump in her left hand which had been present since her 20s, and had recently become painful and pulsatile (Figure 6). MRI demonstrated a small cluster of vessels in the subcutaneous tissue overlying the palmar aspect of the second metacarpal head. MRA did not reveal any feeding arteries or draining veins. Appearances were in keeping with a low-flow PVM, which was supported by normal ASL signal intensity in the region.

## Discussion

This case series provides preliminary evidence on the feasibility and diagnostic performance of ASL in discriminating between high- and low-flow PVMs across a range of anatomical locations. There is a strong qualitative correlation for flow rate between ASL and conventional imaging techniques (particularly gadolinium-enhanced MRA and catheter angiography), suggesting that ASL is reliable in differentiating flow dynamics. This has significant implications for management. For example, transarterial embolisation may be offered for high-flow lesions with the aim of occluding the nidus or fistulous connection. Indeed, accurate embolisation of the nidus has shown better symptomatic improvement and long term prevention of AVM recurrence.^8^ In addition, identifying high-flow lesions is clinically important due to the potential systemic complications, such as high output cardiac failure secondary to excessive arteriovenous shunting.^9^ In contrast, low-flow lesions may be treated with percutaneous sclerotherapy and are usually more straightforward to manage.^2^ ASL imaging could provide a useful screening tool in equivocal cases to exclude any lesional high flow components. Moreover, ASL provided additional information to MRA in two patients (cases 2 and 3) by identifying potential niduses where blood flow is thought to be maximal, and may offer a target for embolization in Case 2. We failed to demonstrate that ASL consistently identified arterial feeders and draining veins in the high-flow lesions, however more research corroborating with catheter angiography is needed in this area.

ASL confers important advantages to existing imaging techniques. Firstly, ASL offers an objective, quantitative measure of flow. Additionally, it allows a more physiological measure as water in arterial blood is used as an endogenous tracer.^10^ Together, these may provide a greater understanding of the flow dynamics of PVMs. Secondly, ASL has the ability to identify the nidus which can facilitate planning for endovascular procedures. This has been highlighted both by Case 2 in this series and the prior case report.^6^ Thirdly, it avoids the need for intravenous gadolinium as used in certain MRA techniques. This in turn can reduce cost, remove the need for an intravenous cannula (advantageous in instances where access is difficult and to improve patient comfort) and provide an alternative imaging technique in the rare circumstances where the administration of gadolinium should be either avoided or reviewed, such as patients at higher risk of hypersensitivity reaction, pregnancy, severe renal impairment and paediatric patients in the first year of life.^11,12^ Finally, there is evidence that ASL is useful in the follow up of intracranial AVMs,^13,14^ with the potential for similar application in high-flow PVMs. For example, this would be beneficial for the patient in Case 3 who is under regular follow up for her AVM and may circumvent the need for repeated gadolinium-enhanced MRA.

Previous experience from the use of ASL in intracranial imaging and the results from this case series demonstrate some of its technical limitations. Firstly, signal-to-noise ratio (SNR), spatial resolution and magnetisation transfer effects may all pose problems in the imaging of PVMs.^15^ The lower spatial resolution of ASL may explain why the small calibre arterial feeder in case one was not delineated. Furthermore, low SNR may be a particular issue in the extremities where blood flow is slower and the imaged body part is smaller, and is highlighted by Case 6 where the region of interest was in the finger. Secondly, the optimal labelling and MR parameters for different anatomical locations have not yet been fully established. Indeed, altering parameters such as post-labelling delay time has been shown to change nidal, venous and gray matter perfusion in intracranial AVMs,^16^ with the potential of PVMs being similarly affected. Thirdly, ASL may underestimate total blood flow in high-flow PVMs, as an average value of flow from the entire region of interest is calculated (including both the high-flow nidus and feeding/draining vessels).^14^ Fourthly, there is a theoretical risk of signal inhomogeneities in certain vascular territories,^17^ depending on the location of the PVM and its arterial supply with respect to the labelling plane. Finally, recovery of spin inversion in arterial water during its transit results in decreased signal from venous drainage,^18^ resulting in potential difficulties in assessing draining veins. However, the limited evidence from this case series suggests relatively reliable performance in delineating large calibre venous drainage.

To conclude, ASL continues to show a promising role in the imaging of PVMs, particularly in discriminating between high- and low-flow lesions. Given the benefits of ASL, future research should focus on larger scale studies to validate and quantitatively establish the diagnostic performance of ASL, and on establishing its limitations in peripheral imaging.

## Learning points

- ASL is a MR perfusion technique where water in arterial blood is used as an endogenous freely diffusible tracer. It has a number of advantages and limitations.
- We provide preliminary evidence for the ability of ASL to distinguish between high- and low-flow PVMs, with important implications for patient management.
- ASL may facilitate characterisation of the nidus in certain high-flow PVMs, which can help plan endovascular therapies.
